# Predicting Vulnerabilities of North American Shorebirds to Climate Change

**DOI:** 10.1371/journal.pone.0108899

**Published:** 2014-09-30

**Authors:** Hector Galbraith, David W. DesRochers, Stephen Brown, J. Michael Reed

**Affiliations:** 1 Manomet Center for Conservation Sciences, Manomet, Massachusetts, United States of America; 2 Department of Natural Sciences, Dalton State College, Dalton, Georgia, United States of America; 3 Department of Biology, Tufts University, Medford, Massachusetts, United States of America; 4 National Wildlife Federation, Springfield, Massachusetts, United States of America; Point Blue Conservation Science, United States of America

## Abstract

Despite an increase in conservation efforts for shorebirds, there are widespread declines of many species of North American shorebirds. We wanted to know whether these declines would be exacerbated by climate change, and whether relatively secure species might become at–risk species. Virtually all of the shorebird species breeding in the USA and Canada are migratory, which means climate change could affect extinction risk via changes on the breeding, wintering, and/or migratory refueling grounds, and that ecological synchronicities could be disrupted at multiple sites. To predict the effects of climate change on shorebird extinction risks, we created a categorical risk model complementary to that used by Partners–in–Flight and the U.S. Shorebird Conservation Plan. The model is based on anticipated changes in breeding, migration, and wintering habitat, degree of dependence on ecological synchronicities, migration distance, and degree of specialization on breeding, migration, or wintering habitat. We evaluated 49 species, and for 3 species we evaluated 2 distinct populations each, and found that 47 (90%) taxa are predicted to experience an increase in risk of extinction. No species was reclassified into a lower–risk category, although 6 species had at least one risk factor decrease in association with climate change. The number of species that changed risk categories in our assessment is sensitive to how much of an effect of climate change is required to cause the shift, but even at its least sensitive, 20 species were at the highest risk category for extinction. Based on our results it appears that shorebirds are likely to be highly vulnerable to climate change. Finally, we discuss both how our approach can be integrated with existing risk assessments and potential future directions for predicting change in extinction risk due to climate change.

## Introduction

Shorebirds are important components of the ecosystems in which they live, they are valued by the general public, can exhibit extremely large and impressive aggregations during migration, and they can act as sentinels of global environmental change [1]–[3]. There also is a growing demand to move beyond evaluating climate change impacts on single species or habitats and to evaluate expected broad scale ecological impacts on communities and ecosystems [4]. Consequently, we are concerned about the current documented widespread declines of many species of North American shorebirds [1], [5]–[8], particularly the recent steep declines in Atlantic populations of Red Knots (scientific names of North American shorebirds are given below) [9]–[10] and Semipalmated Sandpipers [11]–[12].

The U.S. Fish and Wildlife Service currently lists three North American shorebirds as Threatened or Endangered [13]. IUCN lists five shorebird species in North America as Near Threatened or at higher risk, and four additional species in these categories for the Western Hemisphere [14]. The causes of these declines are not well understood but most likely include loss of breeding, migration, and wintering habitats, and disturbance and exploitation [1], [15]–[17]. It should be recognized, however, that the factors causing such changes could be global, since population reductions have been seen in virtually all shorebird flyways from North and South America, to East Africa, to Asia and Australia, e.g., [18]–[19].

Global climate change is an anthropogenic stressor that could adversely affect shorebird populations across species' ranges. Shorebirds that breed and/or winter at high latitudes may be among the most sensitive of bird species to this stressor because this is where climate change is expected to be most severe [20]. They also have several additional risk factors, including lengthy, energetically expensive migrations where they may be vulnerable to changes in wind patterns, dependence upon coastal migration stopover sites that are vulnerable to sea level rise, and dependence upon ecological synchronicities that may be disrupted by a changing climate [16], [21]–[23]. Small–Lorenz *et al.* [24] point out that assessments of vulnerability to climate change often ignore problems associated with a migratory life–history, causing them to underestimate vulnerabilities. Shorebirds are already in a vulnerable condition and climate change may exacerbate this.

If we are to understand what may happen to shorebirds in the near future and initiate appropriate conservation measures it is essential that we be able to predict the likely vulnerabilities of shorebird species to various aspects of the changing climate, cf. [25]. To be useful for conservation, predictive frameworks should be based on the ecologies and life histories of the species, should incorporate what we know about how the planet's climate will alter, and should generate at least qualitative estimates of species vulnerabilities, e.g. [26]–[27].

Categorizing vulnerability to extinction based on a suite of characteristics, such as population size and rate of decline, is used widely e.g. [28]–[31]. The best known models are those of Partners in Flight and IUCN (also used by BirdLife International) [32]. Their categorization approach to vulnerability also can be used to evaluate species' changes in vulnerability as ecosystems change over time, e.g. [33]. Partners in Flight (PIF) uses a model to assess vulnerability based on population trend, relative abundance, threats during the breeding and non–breeding seasons, and breeding and non–breeding range sizes. For each category, each species receives a score of 1 to 5, with 5 associated with greatest risk. These scores are summed using several different formulas, each of which is used to determine species of conservation concern for particular reasons. A similar system was developed based on the same set of basic variables for the U.S. Shorebird Conservation Plan [1], although the resulting risk categories are defined somewhat differently. None of these systems includes risk due to climate change. In this paper, it was our overarching goal to determine the degree to which climate change will alter the extinction risk level assigned to shorebird species in the U.S. Shorebird Conservation Plan, and for this method to be compatible with the PIF ranking system.

We approached our reconsideration of risks under climate change by developing an assessment framework, and then used it to evaluate the vulnerabilities to climate change of North American (north of Mexico) shorebird species, whose life histories extend across wide ecological and behavioral spectra. Specifically, we (1) identified risk factors, (2) created a framework for quantifying the change in risk due to climate change for each of the factors, including the possibility of decreased extinction risk due to climate change, (3) identified the effects climate change would have on the risk factors, (4) reviewed the literature on each shorebird species we assessed to determine species–specific risk for each factor, and (5) assigned species to their new extinction–risk categories. We also (6) did a sensitivity analysis to determine how the results were affected by different decision rules for changing PIF risk categories.

## Methods

We included 49 species in our assessment. For three species (Willet, Piping and Snowy Plover) we evaluated two distinct populations each, so in all 52 taxa were evaluated. We excluded Eskimo Curlew (*Numenius borealis*) from our analysis because it is likely extinct [34]. Our assessments are compatible with both the U.S. Shorebird Conservation Plan and PIF frameworks, although because of the increased risk to some species already at the highest risk categories, we needed to add a new risk category – critical – to distinguish species at greatly increased risk.

To achieve the goal of creating a framework that could be integrated with both PIF and the U.S. Shorebird Conservation Plan, we first evaluated other existing approaches. The State of the Birds [35] developed a framework to assess changes in risk due to climate change, with the goal of applying it to all bird species. They included migration distance and timing as bivariate factors (birds that migrate long distances and use daylight cues = 1; else 0); degree of breeding habitat obligation (high = 1 vs. not = 0); dispersal ability (1 vs. 0); niche specificity (1, 0); reproductive potential (lays one egg per year = 1, else 0); and habitat susceptibility (divided into 3 levels, 2, 1, 0, from highest to lowest susceptibility). Scores were summed to assess overall risk. This approach apparently ignores risks associated with migration and wintering habitat obligation, does not allow for extinction risk to decrease due to climate change, and there is heavy weighting of reproductive potential, which is evaluated on a narrow scale that distinguishes only between one–egg clutches and all other clutch sizes. Also, while reproductive potential may be important for population size recovery following sudden decline, it may be less important with respect to gradual climate change. This approach is applicable to other species included in the PIF prioritization system.

We included six factors in our risk framework, each of which had 3–5 risk levels. Factors included: expected losses or gains in (1) breeding, (2) migration, and (3) non–breeding habitat (4) degree of dependence on ecological synchronicities; (5) migration distance; and (6) degree of habitat specialization (on breeding, migration, and non–breeding grounds). All risk factors were given equal weight in the assessment, and each factor is described in detail below.

### Expected Losses or Gains in Breeding, Migration, and Non–breeding Habitat (1–3)

We accepted that the atmospheric concentrations of greenhouse gases will approximately double (over pre–industrial levels) by the middle to the end of the century [36]–[37]. We then summarized, based on current understanding reported in the literature, the effects climatic change should have on habitats used by our focal shorebirds in the western hemisphere. What follows is our assessment of these changes (designated B1–B5), and brief statements about our confidence in these changes. These approximate confidence levels of >70%, 30–70%, and <30% are modified from the 5–category scale developed by [38] for the Intergovernmental Panel on Climate Change Third Assessment Report. We reduced the number of categories because we did not think the implied precision of 5 levels of confidence was defensible.

#### (B1) Northern hemispheric boreal and arctic areas

Tundra habitat will be reduced in extent as the tree line moves poleward; areas that persist as tundra will become less dominated by graminoids and other low–growth species and will become increasingly dominated by more shrubby species, reducing the habitat value for breeding shorebirds [39]–[47]. Also, the boreal forest will extend its range northward as it replaces tundra, but its southern distribution will contract northwards [37]–[44]. Although it is true that new areas of bare ground are likely to be created by ice cap and glacial recession in high tundra areas, we do not believe that this will result in more habitat for most breeding shorebirds since it will persist as gravel or bouldery moraine for a long period until vegetated and soil–forming processes can occur. Confidence = medium.

Changes in precipitation and evapotranspiration are also likely, but the aggregate effects on tundra hydrology are difficult to predict [48]. Drier overall conditions may be likely, and may reduce food availability during the breeding season [48]. It is unclear how climate change will affect the water balance on tundra breeding habitats due to the complex interaction of several factors, including amounts and timing of precipitation events, timing and extent of spring thaw, depth of the active layer, and erosion events [48]–[53]. While annual rainfall is predicted to increase throughout the breeding range, evapotranspiration is also expected to increase enough to more than offset the effect of increased precipitation [36]. The result may be a loss of some wetland breeding habitat to dryer conditions, but this is unclear. Confidence = medium.

#### (B2) North American Great Plains

Much of the climate modeling that has been performed indicates that these interior grassland regions will become hotter and drier [44], [54]–[57]. This is likely to result in adverse impacts to shorebird species that depend on seasonally or permanently flooded wetlands for their migration stopovers. Confidence = medium

#### (B3) Coastal habitats

Based on IPCC [36] and more recent modeling [58]–[60] we assume that sea levels will rise globally by between 1 and 2 meters, resulting in the loss of coastal shorebird habitats. This applies to North, Central and South America [61], and will be worst in areas with, for example, high tidal amplitudes in shallow lagoons and broad estuaries [62]–[65]. Consequently, we anticipate major loss of coastal wintering habitat for shorebirds, particularly in areas where the land surface is subsiding or accretion rates of intertidal habitats are low (e.g., most Gulf Coast sites) [66]–[67]. If coastal habitats are able to move inland in response to sea level rise, it could offset losses, but at many sites this will be precluded by human infrastructure and interventions [21], [65], [68]–[69]. Confidence = high

#### (B4) Interior South America

Ecological modeling based on climate change models indicates that increased aridification in South America will have the following effects: first it is likely to result in the replacement of currently forested areas in the Amazon by savanna habitat and seasonal forests [70]–[71]. Experimental droughts in the eastern Brazilian Amazon resulted in increased tree mortality, which also supports the expectation of declining rainforest habitat [72]. This is unlikely to benefit shorebirds as few use the existing savanna habitats in central South America. Second, the existing grassland areas in central and southern South America will become drier [36], [73], but the effect on the grassland habitats on which North American shorebirds currently winter is uncertain. Confidence = low.

#### (B5) Eastern North American forests

The only North American shorebird species that primarily uses temperate forest habitat for breeding is the American Woodcock. The species prefers young forest with openings, and the species tolerates a wide a range of tree species [74]. In much of the woodcock's range afforestation is occurring due to ecological succession resulting from abandonment of historical agricultural areas [75]. As a result, young forests adjacent to fields or containing areas of open habitat are declining, resulting in loss of required breeding habitat. Additionally, climate change likely will result in increased vegetation growth at higher latitudes in North America [38]–[41]. This will result in the establishment of more woody vegetation and a subsequent increase in young forest habitat in the north. It is unclear if northward expansion of the woodcock's range is occurring, so changes in forest landscape may outpace range expansion. Another potential concern for forest breeding habitat is climate change's impact on tree mortality. There is growing evidence that drought resulting from climate change leads to increased tree mortality [76]–[78]. This may open breeding areas for American Woodcock locally, but widespread forest loss could result in loss of breeding habitat. Confidence = Medium.

#### (B6) Ocean

One of the primary mechanisms through which climate change could impact oceanic habitats is through acidification [79]–[80]. This likely will reduce the quality of marine habitats, but the extent to which this might affect pelagic non–breeding shorebirds is uncertain [36], [81]–[82]. One hypothesis is that ocean acidification could reduce the fitness of many plankton species by reducing calcification and other physiological processes [83]–[84]. If ocean acidification does negatively impact marine plankton food resources, the decrease could be offset, however, by increased ocean upwelling which could function to increase food resources [85]. Confidence = low.

### Ecological Synchronicities (4)

We recognize two types of ecological synchronicities important to shorebirds that we think could be affected by climate change.

#### (ES1) Breeding season food resources

Arctic temperatures are rising and are projected to further increase in the future, resulting in earlier spring thaws and ice melts [36]. This likely will result in earlier invertebrate hatches because arctic invertebrate emergence is temperature dependent [86]. Long–term field observations and recent experimental warming studies of arctic plots support this hypothesis [87]–[88]. If birds are unable to alter migration timing, then arctic nesting shorebirds may have insufficient food resources for young.

#### (ES2) Migration food resources

Some migrants depend on highly seasonal food sources during migration [89]. For example, shorebirds such as Ruddy Turnstones, Red Knots, Sanderlings, and Semipalmated Sandpipers are highly reliant on American Horseshoe Crab (*Limulus polyphemus*) eggs for refueling during northward migration stopovers [90]–[91]. If climate change affects timing of horseshoe crab breeding, this would disrupt synchronicity between horseshoe crab egg laying and spring migration.

### Migration Distance (5)

**Figure 1:**
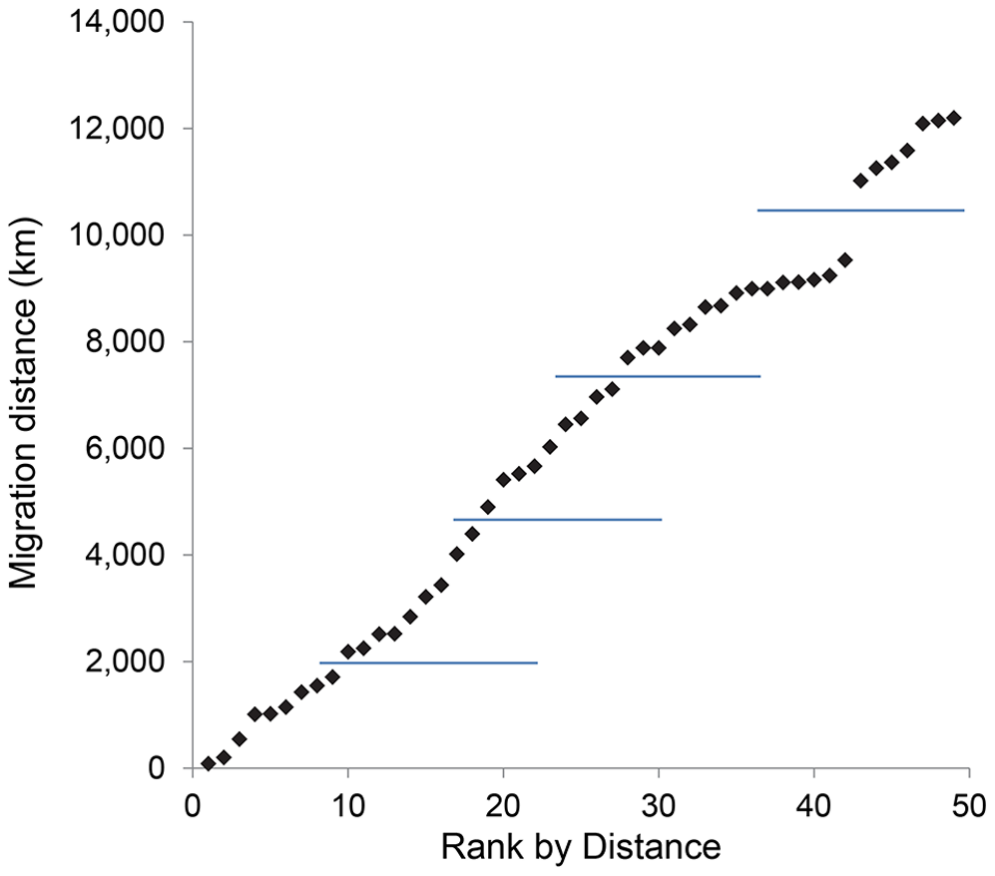
One-way migration distances calculated as mid–point to mid–point of their summer and winter geographic ranges. Ranges were downloaded from the NatureServe database. Horizontal lines separate dispersal distances as ranked in Table 1, with the shortest distances associated with rank 1 and the greatest distances with rank 5. The exceptions are the Bristle–thighed Curlew and Bar–tailed Godwit, which do not overwinter in the New World so they are not covered by the database. They fall into the greatest migration distance category, and are represented arbitrarily in the figure by the 2 points showing the greatest migration distances.

We treat migration distance as others have, as a surrogate for things that can go wrong that have not been captured by other factors [31], [35], [92]. The assumption is that the farther a species has to migrate, the more ecological disruption can occur [92]–[94]. In the context of climate change, for example, migratory connectivity interacts with habitat loss from sea level rise [95]–[96] and species may encounter more severe weather during migration [97]–[98]. Our separation of species into distance categories was done by looking for natural breaks in the migration distance data, resulting in distances being divided into 5 categories (Fig. 1). Migration distances were calculated from the approximate center of each species' breeding range to the approximate center of each species' wintering range using data from NatureServe [99]. The two exceptions were Bristle–thighed Curlew and Bar–tailed Godwit, which are not covered by this database. Known migration distances placed these species in the greatest–distance category.

### Degree of Habitat Specialization (6)

This variable refers to degree of specialization to a certain habitat type, rather than the vulnerability of the habitat type. We assert that being specialized increases your extinction risk to climate change because of reduced response capability. If a species specializes on a habitat type at any time in its life cycle (breeding, migration, non–breeding), it was considered to be specialized. We divided this risk factor into three categories (Table 1).

**Table 1:** List of risk factors evaluated for species sensitivity to climate change.

1) Loss/gain in breeding habitat under climate change:	Score	Arrow
Major losses (>50%)	5	↑↑
Moderate losses (10–50%)	3	↑
Limited or no losses (−10–10%)	0	0
Moderate increase (10–50%)	−3	↓
Major increase (>50%)	−5	↓↓
2) Loss/gain in wintering habitat under climate change:		
Major losses (>50%)	5	↑↑
Moderate losses (10–50%)	3	↑
Limited or no losses (−10–10%)	0	0
Moderate increase (10–50%)	−3	↓
Major increase (>50%)	−5	↓↓
3) Loss/gain in migration habitat under climate change:		
Major losses (>50%)	5	↑↑
Moderate losses (10–50%)	3	↑
Limited or no losses (−10–10%)	0	0
Moderate increas (10–50%)	−3	↓
Major increase (>50%)	−5	↓↓
4) Degree of dependence on ecological synchronicities:		
High	5	↑↑
Moderate	3	↑
Low	0	0
5) Migration distance ( = surrogate for a suite of issues):		
see figure 1 for distance categories	5	↑↑
	4	↑
	3	↑
	2	0
	1	0
6) Degree breeding, wintering or migration habitat specialization		
Highly specialized	5	↑↑
Specialized	4	↑↑
Somewhat specialized	3	↑
Not specialized	0	0

Values are given by scores (similar to the PIF approach) and by arrows. Note that negative scores/down arrows indicate a decreased extinction risk due to climate change. Our current assessment is based on 4 arrows in the same direction (up or down) being sufficient to shift a species to the next risk category.

### Assessment Framework Development

Each risk factor was assessed for each species using information from the literature regarding the natural history of the species and anticipated changes due to climate change. A summary of each species' risk level associated with climate change for each risk factor narrative, as well as confidence scores can be found in Appendices S1 and S2. For each risk factor, for each species, we also included a subjective confidence score (1 = low to 5 = high confidence). We recognize that a species might have increased extinction risk due to climate change, but it might not increase enough to change risk categories.

We described changes in risk using two systems: a numeric scoring system that had maximum values for each factor of 5, and a graphical depiction of the change in risk using arrows because we thought they were more intuitive for rapid visual assessment of changes and patterns. Risk factors were scaled from 0 to 5 to match PIF scaling. For the three habitat factors (1–3), we allowed for the possibility of improved conditions due to climate change. Improvement resulted in negative scores (or down–arrows) to show reduced risk. The factors, and their subdivision and scoring, are shown in Table 1.

For our purposes, we decided that an increase in risk score of 10 (equivalent to 4 ↑s; the arrows indicate the direction and degree of effect) was sufficient to increase by a single risk category because a score of 10 would mean that a species is at extreme risk in two of the six categories. This assignment is a first approximation based on best professional judgment cf. [32], but should be revisited as more information about shorebird ecology and vulnerability to habitat changes becomes available.

To investigate the importance of our decision for how much change in risk is sufficient to cause a change in risk category, we did a sensitivity analysis. Specifically, we assessed the sensitivity of our results – which species were placed into which risk category – to the amount of change in extinction risk that was required for a species to change risk categories. We did this by making the criterion for changing categories more sensitive, requiring the accumulation of only 3 arrows to make the transition between risk categories. We also evaluated the effect of making the criterion less sensitive, evaluating the effects of requiring 5, 6 and 7 arrows to allow a species to change risk categories. If our method is insensitive to this criterion, we would expect little change in categorization with changing criteria.

## Results

Each species' account and changes in risk level are found in Appendices S1 and S2, but we briefly go through the account for the Semipalmated Sandpiper to demonstrate the procedure. (1) We anticipate moderate loss of breeding habitat (score 3; 1 ↑). Our reasoning is based largely on the expectation that tundra breeding habitat will be reduced over the longer term by the increase of woody vegetation, which will invade current areas of tundra [43]. Additional impacts may also occur from changes in precipitation, but it is unclear how climate change will affect the water balance on tundra breeding habitats due to the complex interaction of several factors, including amounts and timing of precipitation, timing of spring thaw, and depth of the active layer [48]. While annual rainfall is predicted to increase throughout the breeding range, evapotranspiration is also expected to increase enough to more than offset the effect of increased precipitation. The result may be a loss of some wetland breeding habitat to dryer conditions, but this is unclear. Our confidence in the assessment of the overall score for moderate loss of breeding habitat is low. (2) We anticipate major loss of wintering habitat (score 5; 2 ↑s) because winter range includes almost exclusively coastal shoreline habitat, so sea level rise (SLR), storm surges, and changing fresh–salt water mixes pose a large threat. Since the species uses estuaries with large tidal amplitudes in Brazil, this may buffer against the SLR impacts, at least locally. Our confidence in this estimate is high. (3) We anticipate moderate loss of migration habitat (score 3; 1 ↑) because SLR likely will cause the loss of some coastal migratory areas. Expected decrease in rainfall in southern areas of North America will cause a decrease in spring migration habitat. In contrast, rainfall is expected to increase in northern portions of North America during spring migration, likely resulting in increased habitat in the interior. Our confidence in this estimate is high. (4) This species has a high degree of dependence on ecological synchronicities (score 5; 2 ↑s). Arctic temperatures are expected to increase, resulting in earlier spring thaws and ice melts. This, in turn, will likely result in earlier invertebrate emergence. If birds are unable to alter migration timing, then arctic nesting shorebirds may have insufficient food resources to support reproduction. Our confidence in this estimate is high. (5) Migration distance is 7886 km (score 4; 2 ↑s). (6) We categorize this species as being moderately specialized in its habitat use (score 4; 2 ↑s). It has fairly specific wintering habitat requirements, including shorelines with wide intertidal mudflats, near shallow lagoons, and wide estuaries with large tidal amplitudes. Our confidence in this estimate is high. This assessment generates a total score of 24 (9 ↑s), which is enough in our protocol to push the species up two risk categories from its place in the current U.S. Shorebird Conservation Plan, from a species of Moderate Concern to Highly Imperiled.

**Figure 2:**
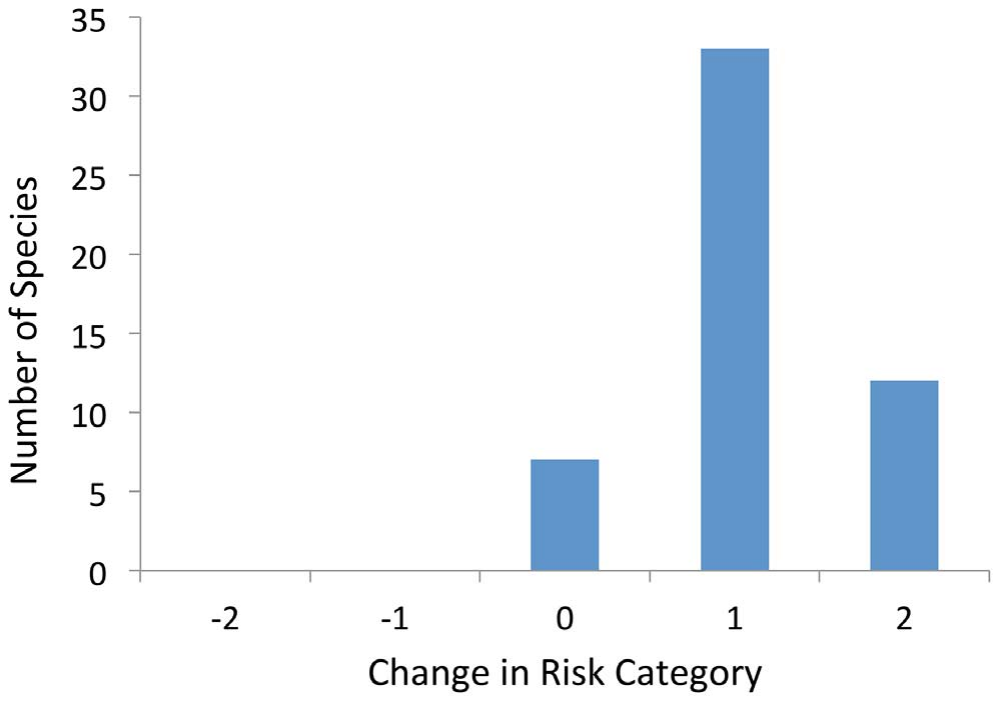
Number of species that we predict will not change U.S. Shorebird Conservation Plan Risk Categories due to climate change (0), and the number that will have increased risk of extinction (positive values); we predicted no species to have reduced risk (negative values). Data are summarized from Table 2 (differences between last two columns).

Of the 52 taxa we evaluated, 45 (87%) are predicted to qualitatively increase their risks of extinction as a result of climate change; 33 by one level in the U.S. Shorebird Conservation Plan, and 12 by 2 levels (Table 2, Fig. 2). Only three species had risk factors that we predict will lower a species' extinction risk due to climate change: Solitary Sandpiper, due to the creation of more breeding habitat; Bristle–thighed Curlew, due to the expansion of breeding and wintering habitat; and White–rumped Sandpiper, due to more wintering habitat. The U.S. Shorebird Conservation Plan currently lists 29 species at risk levels of High Concern or higher, and no species are considered Not at Risk. Based on our assessments, we categorize 43 taxa (species+races, hereafter ‘species’ or ‘taxa’) as High Concern or higher due to increased risks resulting from climate change, with 15 of these being in the newly created Critical category (Table 2).

**Table 2:** Results of predicted change in extinction risk to shorebird species based on climate change.

Common name	Scientific name	Breeding habitat	Wintering habitat	Migration habitat	Ecological synchronicity	Migration distance	Degree of habitat specialization	Sum of change (arrows)	State of the Birds 2010^1^	Current risk category	Revised risk category^2^
Black–necked Stilt	*Himantopus mexicanus*	↑↑	↑	↑	0	0	↑↑	6	0	2	3
American Avocet	*Recurvirostra americana*	↑↑	↑↑	↑↑	0	0	↑	7	1	3	4
American Oystercatcher	*Haematopus palliatus*	↑↑	↑↑	↑↑	0	0	↑↑	8	4	4	6
Black Oystercatcher	*Haematopus bachmani*	↑	↑	↑	0	0	↑↑	5	4	4	5
Black–bellied Plover	*Pluvialis squatarola*	↑↑	↑	↑	↑	↑	↑↑	8	3	3	5
American Golden–Plover	*Pluvialis dominica*	↑↑	↑	↑	↑	↑↑	↑	8	2	4	6
Pacific Golden–Plover	*Pluvialis fulva*	↑↑	0	0	↑	0	↑↑	5	3	4	5
Snowy Plover – coastal	*Charadrius nivosus*	↑↑	↑↑	↑↑	0	0	↑↑	8	2	5	6
Snowy Plover – inland		↑	↑	↑↑	0	0	↑↑	6	2	5	6
Wilson's Plover	*Charadrius wilsonia*	↑↑	↑↑	↑↑	0	0	↑↑	8	3	4	6
Semipalmated Plover	*Charadrius semipalmatus*	↑	↑	↑	↑	↑	↑↑	7	1	2	3
Piping Plover – coastal	*Charadrius melodus*	↑↑	↑↑	↑↑	0	0	↑↑	8	3	5	6
Piping Plover – inland		↑↑	↑↑	↑	0	0	↑↑	7	3	5	6
Killdeer	*Charadrius vociferous*	0	0	0	0	0	0	0	0	3	3
Mountain Plover	*Charadrius montanus*	↓	↑	↑	0	0	↑↑	3	1	5	5
Spotted Sandpiper	*Actitis macularius*	0	0	0	0	↑	0	1	1	2	2
Solitary Sandpiper	*Tringa solitaria*	↓	↑↑	↑↑	↑	↑	↑↑	7	2	4	5
Wandering Tattler	*Tringa incana*	0	↑	0	↑	↑	↑	4	4	3	4
Greater Yellowlegs	*Tringa melanoleuca*	0	0	0	↑	↑	↑↑	4	1	3	4
Willet – eastern	*Tringa semipalmata*	↑↑	↑↑	↑↑	0	0	↑	7	2	3	4
Willet – western		↑	↑	↑↑	0	0	↑	5	2	3	4
Lesser Yellowlegs	*Tringa flavipes*	↓	↑	↑	↑	↑	↑	4	1	3	4
Upland Sandpiper	*Bartramia longicauda*	0	0	0	0	↑	↑↑	3	2	4	4
Whimbrel	*Numenius phaeopus*	↑↑	↑↑	↑↑	↑	↑	↑	9	3	4	6
Bristle–thighed Curlew	*Numenius tahitiensis*	↓	↑↑	↑	↑	↑↑	↑	6	4	4	5
Long–billed Curlew	*Numenius americanus*	↑↑	↑	0	0	0	↑↑	5	2	5	6
Hudsonian Godwit	*Limosa haemastica*	0	↑	0	↑	↑↑	0	4	4	4	5
Bar–tailed Godwit	*Limosa lapponica*	↑↑	↑↑	↑↑	↑	↑↑	↑↑	11	4	4	6
Marbled Godwit	*Limosa fedoa*	↑	↑	↑	0	0	↑↑	5	1	4	5
Ruddy Turnstone	*Arenaria interpres*	↑	↑↑	↑↑	↑↑	↑	↑↑	10	3	4	6
Black Turnstone	*Arenaria melanocephala*	↑	↑	↑	↑	0	↑↑	6	4	4	5
Red Knot	*Calidris canutus*	↑	↑↑	↑↑	↑↑	↑	↑↑	10	4	4	6
Surfbird	*Calidris virgata*	↑	↑↑	↑↑	↑	↑	↑↑	9	5	4	6
Stilt Sandpiper	*Calidris himantopus*	↑↑	0	0	↑	↑	↑↑	6	3	3	4
Sanderling	*Calidris alba*	↑↑	↑↑	↑↑	↑↑	↑	↑↑	11	3	4	6
Dunlin	*Calidris alpina*	↑↑	↑↑	↑	↑	0	↑↑	8	3	3	5
Rock Sandpiper	*Calidris ptilocnemis*	↑	0	0	↑	0	↑↑	4	4	3	4
Purple Sandpiper	*Calidris maritima*	↑	0	0	0	0	↑↑	3	4	2	2
Baird's Sandpiper	*Calidris bairdii*	0	0	0	↑	↑↑	↑↑	5	3	2	3
Least Sandpiper	*Calidris minutilla*	0	↑	↑	↑	↑	↑	5	3	3	4
White–rumped Sandpiper	*Calidris fuscicollis*	↑↑	↓	↑	↑	↑↑	↑	6	3	2	3
Buff–breasted Sandpiper	*Calidris subruficollis*	↑↑	0	0	↑	↑↑	↑↑	7	3	4	5
Pectoral Sandpiper	*Calidris melanotos*	↑↑	0	0	↑	↑	↑↑	6	3	2	3
Semipalmated Sandpiper	*Calidris pusilla*	↑↑	↑↑	↑	↑↑	↑	↑↑	10	4	3	5
Western Sandpiper	*Calidris mauri*	↑	↑↑	↑	↑	↑	↑	7	4	4	5
Short–billed Dowitcher	*Limnodromus griseus*	0	↑↑	↑↑	↑	↑	↑	7	4	4	5
Long–billed Dowitcher	*Limnodromus scolopaceus*	↑↑	↑	↑	↑	↑	↑	7	3	4	5
Wilson's Snipe	*Gallinago delicata*	0	0	0	0	↑	↑	2	0	3	3
American Woodcock	*Scolopax minor*	↑	↓	↓	0	0	↑↑	1	1	4	4
Wilson's Phalarope	*Phalaropus tricolor*	↑	↑	↑	0	↑	0	4	3	4	5
Red–necked Phalarope	*Phalaropus lobatus*	↑↑	0	0	↑	↑	↑↑	6	1	3	4
Red Phalarope	*Phalaropus fulicarius*	↑↑	0	0	↑	↑	↑↑	6	3	3	4

Arrows depict extent and direction of change in risk associated with climate change. See Table 1 for description of risk factors and scoring, and see Appendices S1 and S2 for species specific discussion. Also included in current U.S. Shorebird Conservation Plan (USSCP) Risk Categories and State of the Birds vulnerability score and our proposed revised USSCP risk categories based on the added effects of climate change. Our Assessment of change in risk due to climate change is assessed by adding arrows across rows (1 up arrow and 1 down arrow result in a net of 0 arrows), and using the decision rule of 4 arrows (net, up or down) to shift risk categories.

1 The lower scores, which were not published as part of the State of the Birds, were provided by the U.S. NABCI Committee.

2 Based on our risk analysis, this would be the new U.S. Shorebird Conservation Plan risk category; we added a new risk category – 6 (Critical, Table 3) – to account for qualitatively greater risk than the current U.S. Shorebird Conservation Plan assessment allows.

Of the 52 taxa assessed, 38 (73%) showed increased vulnerabilities due to effects of climate change on breeding habitat, 36 (69%) due to effects on wintering habitat, and 34 (65%) due to migration habitat (Table 2). More taxa also exhibited maximal negative responses (criteria in Table 1) to climate change on the breeding grounds than to winter or migration habitat (24 taxa vs. 19 and 16, respectively). That is, more taxa exhibited increased risk due to climate change on the breeding grounds than for the wintering and migration grounds, and the risks were higher. The number of taxa predicted to have no response or a positive response to climate change was similar across breeding, winter, and migration habitat (13, 15, and 18 taxa respectively). Ecological synchronicity and migration distance, by comparison, had less of an effect on extinction risk due to climate change, with 17 (33%) and 14 (27%) species, respectively, showing no negative effect due to climate change. The greatest risk factor of those assessed, however, was degree of habitat specialization, with 47 (90%) of the taxa showing a negative response to climate change (Table 2).

A natural potential comparison of our results is with those of the State of the Birds [35]. This is a somewhat difficult comparison to make, however, because we used different scales for our risk categories. However, there appears to be general, qualitative concordance for many species. For example, of the 12 species where they predict no (0 score out of 5) or a low (1 score) increase of extinction risk due to climate change, we predict no or low effects on all of them; i.e., our results leave the species in the same risk category or increase by one category (Table 2). However, we predict an increase of only a single risk category on an additional 19 species where State of the Birds predicts greater impacts of climate change (scores of ≥2). Although there is a lot of variability, our results are generally, but not closely, consistent with those of the State of the Birds (r^2^ = 0.27). Our biggest difference occurs for the Purple Sandpiper, where we predict no change in risk category due to climate change, while State of the Birds predicts a strong response (score = 4). Although not to the same degree, we also predict substantially lower increases in extinction risk due to climate change for Black Oystercatcher, Wandering Tattler, Bristle–thighed Curlew, Hudsonian Godwit, Surfbird, Western Sandpiper, and Rock Sandpiper (Table 2).

The number of species that change risk categories in our assessment was sensitive to how much of an effect of climate change is required to cause the shift (Table 3; Appendices S3 and S4). When we make it easier to shift categories (3 arrows to change), we are left with only five species in the moderate or lower concern categories and 22 species in the highest (newly created ‘critical’) risk category, compared to 9 and 15, respectively, when 4 arrows are required to change categories. There is less sensitivity in the other direction. Even when we require 7 arrows to change risk category, we still have 20 species in the highly imperiled or critical risk categories, compared to only 6 when climate change is not considered (Table 3; Appendices S3 and S4). Consequently, one might argue about the most appropriate degree of increased risk required to change risk categories; however, regardless of the threshold used, we conclude that there is an important shift in the numbers of North American shorebirds species at risk of extinction due to climate change.

**Table 3:** Results of sensitivity analysis of risk categorization for shorebird species.

		Projected under with climate change, from most (7) to least (3) conservative transition criterion				
Risk Category	Current	7↑	6↑	5↑	4↑	3↑
Not at risk	0	0	0	0	0	0
Low concern	7	6	3	2	2	1
Moderate concern	16	12	12	11	7	4
High concern	23	14	13	13	13	11
Highly imperiled	6	17	18	17	15	14
Critical	–1	3	6	9	15	22

What is shown in the first column of results is the current distribution of taxa across risk categories by the U.S. Shorebird Conservation Plan (USSCP). The columns that follow are the predicted distributions under different criteria for changing risk category. In Table 2 we assume that the accumulation of 4 arrows across risk factors is sufficient for a species to change risk category; this table shows the sensitivity of this result using more liberal (3 arrows) and more conservative (5, 6, and 7 arrows) criteria for changing risk category. We added a new risk category to those used by Partners–in–Flight (PIF) and the USSCP, Critical, to account for species being at categorically greater risk than previously considered. (See Table 2 and Appendices S3 and S4 for species–specific assessments and summaries.)

1 Category does not exist in current PIF framework.

## Discussion

Many species of shorebirds are the focus of conservation efforts aimed at reversing population declines e.g. [9], [100], so there is a need to prioritize conservation actions that can have the largest impact on the species most in need. The system currently in use for prioritizing shorebird conservation efforts in the United States was developed in 1999–2001 [1], and did not explicitly include vulnerability to the impacts of a changing climate, e.g. [101]. Many studies have shown that climate change poses risks to populations of plants and animals and that impacts to vulnerable species are already occurring, e.g. [102]–[104]. It is expected that such adverse impacts will become more severe and widespread in the future as the climate continues to change. One major application of the system developed in the present study would be to revise the priority scores given to shorebird species by updating the threat scores with the information presented here regarding vulnerability to climate change. We recommend that the U.S. Fish and Wildlife Service revise shorebird priority scores as suggested here, so that the impacts of a changing climate can be more fully integrated into efforts to conserve shorebirds. In addition to applying this information to shorebird species, the same approach could also be applied to other birds. The Partners–in–Flight prioritization system also could be updated to include the approach presented here, if the information on relative risks were collected for other species. This would allow a similar update to reflect vulnerability to climate change across a wide range of bird taxa. We do note that the species assessments and criteria assigned in this manuscript should be considered as first approximations, and will undoubtedly be revised with further discussion by a wider audience. Our primary goal was to establish a system for evaluating the increased risk to species from climate change with respect to existing threat assessments, and to start a discussion about the appropriate values for various species.

Shorebird populations and flyways across the planet are currently being affected by other stressors, many of them unknown, in addition to climate change, e.g., [5]. These impacts are resulting in severe population reductions [1], [6]–[8]. Based on our analyses, adding the stresses and risks imposed by a changing climate to this already threatened baseline renders shorebirds even more vulnerable to extinction. If we are correctly to understand the risks to which shorebirds are exposed, and to identify and implement effective conservation strategies and actions, it is important that we understand these vulnerabilities, particularly those that will occur due to climate change. The purpose of this study was to assess the climate change risks to shorebirds and incorporate these into existing vulnerability evaluations so that we gain a better understanding of the entire panoply of risk factors to which these species are exposed, and their resulting overall vulnerabilities.

Based on our results it appears that shorebirds, as a group, are likely to be highly vulnerable to the changing climate. These vulnerabilities are due to a number of factors. First, many species breed, migrate through, or winter in areas that are likely to be severely impacted by climate change (particularly arctic tundra, coastal breeding, and wintering, and migration stopover sites). Second, the extensive migrations that many of them undertake expose them to risks of changing weather patterns (increased frequencies and intensities of hurricanes, for example) [98]. Shorebirds that require particular staging areas might be more vulnerable to climate change than are those species using stopover sites [95], [105]. Lastly, the ecological synchronicities that many shorebirds depend on (e.g., the complementary timing of the arctic snowmelt and invertebrate prey availability) might suffer disruptions [16], [21]–[24]. Our results reflect these vulnerabilities.

Of the 52 shorebird taxa (49 species, 3 split into 2 populations) that breed in North America and that we evaluated, 45 (87%) were predicted to exhibit an increased extinction risk when the risks posed by climate change were added to their current vulnerabilities as estimated in the U.S. Shorebird Conservation Plan [1]. No species was reclassified into a lower–risk category, although prior to the analysis it had been a possibility. The factors responsible for these increased vulnerabilities were risks of: loss of breeding habitat (particularly for arctic– and coastal–breeders); loss of coastal and inland migration stopover habitats due to sea level rise and drought; and loss of coastal wintering habitat due to sea level rise. Of particular note, for high-Arctic breeders, there is minimal latitude and land for northward range expansion. Extreme weather events were also projected to increase vulnerabilities due to negative effects on habitat, migration mortality, and disruption of ecological synchronicities, e.g. [94].

The increased vulnerabilities of 10 species could not be accommodated using the existing PIF scoring system and we had to create an even higher level of risk than is currently available. These Critical species (including coastal Snowy, Piping, and Wilson's plovers, and inland species such as Mountain Plover and Long–billed Curlew) are already at a high risk level due to other stressors (particularly anthropogenic habitat destruction) and their populations are already declining and jeopardized [32]. The addition of climate change to their risk factors raises them to an even higher level of vulnerability, which may pose even higher threats to their continued existence.

Also of concern is that the addition of climate change to the vulnerability calculations elevates another 18 species to the highest U.S. Shorebird Conservation Plan risk category. Thus, a total of 28 of 49 species are now at the highest risk category under the U.S. Shorebird Conservation Plan, or they exceeded this risk level and had to have an additional category created. The degree to which species changed risk categories was sensitive to our rules of category change. To some degree, as with population viability analyses using stochastic simulation models, which rule we use for category change is a value judgment [106]. Regardless of what rules are used, however, our analysis suggests that shorebirds will have increased vulnerability under climate change, perhaps to a large extent.

Our assessment of extinction risk might be criticized because it does not allow for adaptive capacity in shorebird populations. That is, shorebirds might modify their breeding, migratory, and/or wintering habitat use, foraging, and/or timing to accommodate the changing climate. We already know that some shorebirds in Western Europe have apparently truncated their fall migrations to winter in the Baltic, rather than in oceanic Atlantic countries, such as the UK [104]. Previously, the winter conditions in the Baltic were so harsh that birds had to move farther to exploit the milder conditions of the UK, Holland, etc. Thus, the ameliorating winter conditions in the Baltic have encouraged changes in migration distance [104]. Similarly, In North America, some migratory populations of Hudsonian Godwits have advanced their timing of migration during warm periods, which allows their breeding to synchronize with peak food abundance, while other populations have not [107]. There also is some evidence that Semipalmated and Pectoral sandpipers and Red–necked and Red phalaropes have been observed breeding earlier during warm years [108]. As another example of adapting to changing conditions, Dunlin nestlings can exhibit accelerated growth during periods of low food availability during warm conditions [109]. However, it would be unwise of us to assume that such adaptive capacities were likely to apply across all shorebird species because there is evidence that high Arctic shorebird species may have little capacity for adaptation due to low genetic variability resulting from bottleneck events from previous climate shifts [110]. Time constraints can also cause conflicts among competing life–history requirements, as has been reported in Pied Flycatchers *Ficedula hypoleuca* [111]. Clearly more research needs to be done to determine the degree to which climate adaptation might occur in shorebirds.

What would it take to accurately and precisely predict change in extinction risk due to climate change for migratory shorebirds, or for any species, rather than taking the relatively coarse approach we did in this paper? Certainly there have been detailed assessments of expected regional changes in shorebird populations in response to climate change [112]–[113], and one could create models to link species to landscapes via simulation. But what would be required for accurate, reliable predictions? Strictly speaking, to build a convincing case for an accurate prediction, the first thing we would need is accurate models of climate change. Although there are many models of climate change, and they agree in general with climate trends, there is still a great deal of uncertainty in the exact amount of changes in expected temperature and precipitation, e.g. [114]–[115], particularly at the fine geographic scale that would be needed to understand biotic responses, including the effects of changes in wind patterns [116]. Because hydrological models are complex (i.e., non–linear, with feedback and chaotic dynamics), more accurate data are unlikely to improve model predictions [117]. In addition, accurate regional and local downscaling of global climate models might not be possible [118]–[120].

**Figure 3:**
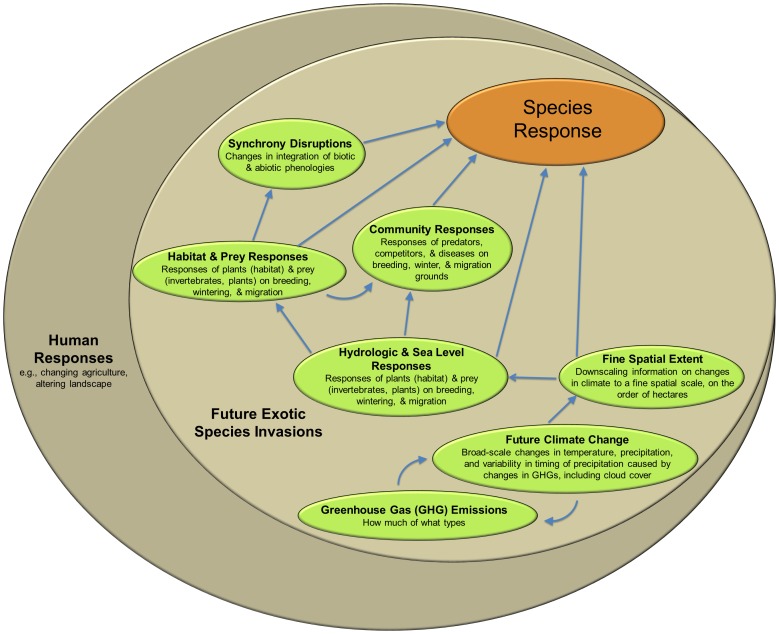
Digraph showing relationships (arrows) for which we need accurate information in order to accurately predict species–specific shorebird responses to climate change. By accurate, we mean variation explained between nodes is >90% or near that, not merely determining statistically significant relationships. Subheadings specify the relationships, and ‘species response’ includes adaptive responses as well as non–adaptive responses. ‘Fine spatial extent’ refers to downscaling climate change estimates to the spatial scale at which species respond; factors at this scale affect species' responses directly and indirectly. The digraph is nested within the contexts of future introductions of exotic, invasive species, and human responses to climate change to indicate that all of the relationships from the digraph can be affected by these particular occurrences or responses.

The next requirement is accurate models linking climate change to hydrologic responses, so we could accurately determine changes in hydrology, amount of sea level rise, the degree to which plant communities will change in response to climate change, in both inland and coastal regions,. Accurate models that allow these predictions do not exist [117], [121]–[123]. Even if we had accurately developed models, we also would need accurate assessments of species' ranges as well as niche–based models for each species we want to evaluate that accurately predicts, with a very high level of variability in distribution explained, the distribution of species, cf. [106]. We do not yet have these, e.g. [124]–[127], and it is not clear to what extent or rate different bird might respond behaviorally to climate change [128]. Finally, we need models that accurately depict community–wide biotic responses to climate change, including accurate anticipation of inter–specific interactions, how local species invasions and extinctions will affect resource availability, how they might change as niches shift [129]–[133]. We do not have these either, and we might be unlikely to accurately anticipate shifting realized niches for a variety of practical reasons [134]–[136]. These challenges are exacerbated by migration because the relationships must be known in breeding, non–breeding, and migration habitats [137]. These relationships we just described are depicted in Fig. 3. Even the highly restrictive requirements we just presented might ultimately be insufficient, because they do not take into account human responses to climate change. For example, what will be the human responses in changes to agricultural practices, relocation away from coastal areas, and so–called adaptive response measures, e.g. [138]–[140], and how will they affect the capacity for ecosystems and shorebirds to respond?

Consequently, we suspect that detailed regional and local biological forecasting of the effects of climate change, even if the correct (but currently unknown) IPCC scenario is selected, is likely to be only generally accurate. Therefore, we think that the relatively coarse assessment of changes in extinction risk that we present here is a useful level of assessment for species at a continental scale; see [31] for another example of a categorical risk assessment at a smaller geographic scale. We stress that the somewhat bleak picture we paint regarding prediction accuracy at small spatial scales should not be used as an excuse to not make models or predictions, or to avoid planning for climate change. Rather, we encourage model development and testing, followed by model revision as more data become available. As with all models, we suggest treating the structure, parameter values, and predictions as hypotheses to test. We also support alternative modeling approaches that might be effective at accommodating model uncertainty, such as robust decision-making [141].

## Supporting Information

Appendix S1 **Vulnerability scores and associated confidence levels for 49 North American breeding shorebird species**
(DOC)

Appendix S2 **Degree of habitat specialization described for each of the taxa**
(DOC)

Appendix S3 **Sensitivity analysis of risk category in which shorebirds are placed**
(DOC)

Appendix S4 **Species in each of the risk categories under the current system, and revised based on climate change.**
(DOC)
