# Supplementary material for: Predicting Vulnerabilities of North American Shorebirds to Climate Change
Source: PLoS One. 2014 Sep 30;9(9):e108899. doi: 10.1371/journal.pone.0108899 (PMC4182597; doi:10.1371/journal.pone.0108899)
Supplement: Appendix S1 — Vulnerability scores and associated confidence levels for 49 North American breeding shorebird species (DOC) [file pone.0108899.s001.doc]

**Galbraith et al.**

**Supplemental material**

| **Appendix 1**  Vulnerability scores and associated confidence levels for 49 North American breeding shorebird species regarding anticipated climate change impacts on habitat used during breeding, winter, and migration along with anticipated impacts on the timing of ecological events. (see Appendix 2 for habitat specificity scores and descriptions.) Vulnerability and confidence scores also are provided for migratory distance. See Birds of North America accounts for primary literature citations regarding habitat use information. Loss/gain of habitat risk factors: Major losses (>50%) = 5, Moderate losses (10–50%) = 3, Limited or no losses/increases (-10–10%) = 0, Moderate increase (10–50%) = -3, Major increase (>50%) = -5. Climate change impact habitat codes (see text for explanation): B1 = Northern hemispheric arctic areas, B2 = Great Plains States and Prairie Provinces in North America, B3 = Coastal habitats, B4 = Interior South America, B5 = forests in eastern North America, B6 = Ocean. Dependence on ecological synchronicity risk factors: High = 5, Moderate = 3, Low = 0; Ecological Synchronicity codes: ES1 = Earlier insect hatches in the Arctic, ES2 = Changes in the timing of horseshoe crab egg availability. | | | | | |
| --- | --- | --- | --- | --- | --- |
| Common name | Breeding habitat | Wintering habitat | Migration habitat | Ecological synchronicity | Migration distance (km) |
| Black-necked Stilt | 5 (High) B2 | 3 (High) B3 | 3 (High) B3 | 0 (High) | 1,010 |
| American Avocet | 5 (High) B2 | 5 (High) B3 | 5 (High) B2-B3 | 0 (High) | 2,185 |
| American Oystercatcher | 5 (High) B3 | 5 (High) B3 | 5 (High) B3 | 0 (High) | 547 |
| Black Oystercatcher | 3 (High) B3 | 3 (High) B3 | 3 (High) B3 | 0 (High) | 84 |
| Black-bellied Plover | 5 (High) B1 | 3 (High) B3 | 3 (High) B3 | 3 (High) ES1 | 6,562 |
| American Golden-Plover | 5 (High) B1 | 3 (Medium) B4 | 3 (Medium) B4 | 3 (High) ES1 | 11,258 |
| Pacific Golden-Plover | 5 (High) B1 | 0 (High) | 0 (High) | 3 (High) ES1 | 4,396 |
| Snowy Plover - coastal | 5 (High) B3 | 5 (High) B3 | 5 (High) B3 | 0 (High) | 1,145 |
| Snowy Plover - inland | 3 (High) B2 | 3 (High) B3 | 5 (High) B3 | 0 (High) | 1,145 |
| Wilson's Plover | 5 (High) B3 | 5 (High) B3 | 5 (High) B3 | 0 (High) | 203 |
| Semipalmated Plover | 3 (High) B1 | 3 (High) B3 | 3 (High) B3 | 3 (High) ES1 | 7,886 |
| Piping Plover - coastal | 5 (High) B3 | 5 (High) B3 | 5 (High) B3 | 0 (High) | 2,522 |
| Piping Plover - inland | 5 (Medium) B2 | 5 (High) B2 | 3 (High) B2 | 0 (High) | 2,522 |
| Killdeer | 0 (High) B2-B3 | 0 (High) B2-B3 | 0 (High) B2-B3 | 0 (High) | 1,549 |
| Mountain Plover | -3 (Medium) B2 | 3 (Medium) B2 | 3 (Medium) B2 | 0 (High) | 1,427 |
| Spotted Sandpiper | 0 (High) B2 | 0 (High) B2-B3 | 0 (High) B2-B3 | 0 (High) | 7,112 |
| Solitary Sandpiper | -3 (High) B1 | 5 (High) B3-B4 | 5 (High) B3-B4 | 3 (High) ES1 | 8,323 |
| Wandering Tattler | 0 (High) B1 | 3 (High) B3 | 0 (High) B3 | 3 (High) ES1 | 5,412 |
| Greater Yellowlegs | 0 (High) B1 | 0 (High) B2-B3 | 0 (High) B2-B3 | 3 (High) ES1 | 7,703 |
| Willet – eastern | 5 (High) B3 | 5 (High) B3 | 5 (High) B3 | 0 (High) | 3,434 |
| Willet – western | 3 (High) B2 | 3 (High) B3 | 5 (Low) B3 | 0 (High) | 3,434 |
| Lesser Yellowlegs | -3 (High) B1 | 3 (High) B4-B5 | 3 (High) B2-B4 | 3 (High) ES1 | 9,164 |
| Upland Sandpiper | 0 (Medium) B2 | 0 (Medium) B4 | 0 (Medium) B2,B4 | 0 (High) | 9,119 |
| Whimbrel | 5 (High) B1 | 5 (High) B3 | 5 (High) B3 | 3 (High) ES1 | 9,114 |
| Bristle-thighed Curlew | -3 (High) B1 | 5 (High) B3 | 3 (High) B3 | 3 (High) ES1 | >8,500 |
| Long-billed Curlew | 5 (High) B2 | 3 (High) B3 | 0 (High) B2-B3 | 0 (High) | 2,250 |
| Hudsonian Godwit | 0 (High) B1 | 3 (High) B3 | 0 (High) B2-B4 | 3 (High) ES1 | 12,095 |
| Bar-tailed Godwit | 5 (High) B1 | 5 (High) B3 | 5 (High) B3 | 3 (High) ES1 | 11,000 |
| Marbled Godwit | 3 (High) B2 | 3 (High) B3 | 3 (High) B2-B3 | 0 (High) | 2,516 |
| Ruddy Turnstone | 3 (High) B1 | 5 (High) B3 | 5 (High) B3 | 5 (High) ES1-ES2 | 8,249 |
| Black Turnstone | 3 (High) B1 | 3 (High) B3 | 3 (High) B3 | 3 (High) ES1 | 2,841 |
| Red Knot | 3 (Low) B1 | 5 (High) B3 | 5 (High) B3 | 5 (High) ES1-ES2 | 8,677 |
| Surfbird | 3 (High) B1 | 5 (High) B3 | 5 (High) B3 | 3 (High) ES1 | 4,900 |
| Stilt Sandpiper | 5 (High) B1 | 0 (High) B4 | 0 (High) B4 | 3 (High) ES1 | 9,243 |
| Sanderling | 5 (High) B1 | 5 (High) B3 | 5 (High) B2-B3 | 5 (High) ES1-ES2 | 6,965 |
| Dunlin | 5 (High) B1 | 5 (High) B3 | 3 (High) B3 | 3 (High) ES1 | 4,018 |
| Rock Sandpiper | 3 (Medium) B1 | 0 (High) B3 | 0 (High) B3 | 3 (High) ES1 | 1,711 |
| Purple Sandpiper | 3 (High) B1 | 0 (High) B3 | 0 (High) B3 | 0 (Low) | 3,214 |
| Baird's Sandpiper | 0 (High) B1 | 0 (High) B4 | 0 (High) B2,B4 | 3 (High) ES1 | 11,022 |
| Least Sandpiper | 0 (High) B1 | 3 (High) B3-B4 | 3 (High) B3-B4 | 3 (High) ES1 | 6,451 |
| White-rumped Sandpiper | 5 (High) B1 | -3 (High) B4 | 3 (High) B4 | 3 (High) ES1 | 11,589 |
| Buff-breasted Sandpiper | 5 (High) B1 | 0 (Medium) B4 | 0 (Medium) B2,B4 | 3 (High) ES1 | 11,366 |
| Pectoral Sandpiper | 5 (High) B1 | 0 (High) B4 | 0 (High) B2, B4 | 3 (High) ES1 | 9,534 |
| Semipalmated Sandpiper | 5 (High) B1 | 5 (High) B3 | 3 (High) B2-B3 | 5 (High) ES1-ES2 | 7,886 |
| Western Sandpiper | 3 (High) B1 | 5 (High) B3 | 3 (High) B2-B3 | 3 (High) ES1 | 6,029 |
| Short-billed Dowitcher | 0 (High) B1 | 5 (High) B3 | 5 (High) B3 | 3 (High) ES1 | 5,524 |
| Long-billed Dowitcher | 5 (High) B1 | 3 (High) B3 | 3 (High) B2 | 3 (High) ES1 | 5,666 |
| Wilson's Snipe | 0 (Medium) B1-B2 | 0 (Medium) B2-B4 | 0 (Medium) B2-B4 | 0 (high) | 8,997 |
| American Woodcock | 3 (Medium) B5 | -3 (High) B5 | -3 (High) B5 | 0 (High) | 1,020 |
| Wilson's Phalarope | 3 (High) B2 | 3 (High) B4 | 3 (High) B4 | 0 (High) | 8,977 |
| Red-necked Phalarope | 5 (High) B1 | 0 (Low) B6 | 0 (Low) B3,B6 | 3 (High) ES1 | 8,914 |
| Red Phalarope | 5 (High) B1 | 0 (Low) B6 | 0 (Low) B3,B6 | 3 (High) ES1 | 8,652 |
