# Supplementary material for: Predicting Vulnerabilities of North American Shorebirds to Climate Change
Source: PLoS One. 2014 Sep 30;9(9):e108899. doi: 10.1371/journal.pone.0108899 (PMC4182597; doi:10.1371/journal.pone.0108899)
Supplement: Appendix S2 — Degree of habitat specialization described for each of the taxa (DOC) [file pone.0108899.s002.doc]

**Galbraith et al.**

**Supplemental material**

| **Appendix 2.** Degree of habitat specialization for 49 North American breeding shorebird species. Vulnerability scores: Highly specialized = 5, Specialized = 4, Somewhat specialized = 3, Not specialized = 0. All confidence scores are high except for Hudsonian Godwit which is medium due to the lack of information on specific habitat requirements during the breeding season. See text for vulnerability score descriptions. | |
| --- | --- |
| Common name | Degree of habitat specialization |
| Black-necked Stilt | 4 – Breeding habitat is specialized; habitats used throughout the remainder of the year are somewhat variable. |
| American Avocet | 3 – Breeding habitat is moderately specialized; habitats used throughout the remainder of the year are somewhat variable. |
| American Oystercatcher | 5 – Highly specific habitat used year-round. |
| Black Oystercatcher | 5 – Highly specific habitat used year-round. |
| Black-bellied Plover | 4 – The species uses a wide variety of habitats throughout the year; habitat use during breeding season is more specialized. |
| American Golden-Plover | 3 – Breeding habitat is specialized; migration and wintering habitats include a range of grassland types. |
| Pacific Golden-Plover | 4 – Breeding habitat is specialized; habitats used throughout the remainder of the year are highly variable. |
| Snowy Plover - coastal | 4 – Breeding habitat is specialized; habitats used throughout the remainder of the year are highly variable. |
| Snowy Plover - inland | 4 – Breeding habitat is specialized; habitats used throughout the remainder of the year are highly variable. |
| Wilson's Plover | 5 – Highly specific habitat used year-round. |
| Semipalmated Plover | 3 – There is some flexibility in breeding habitat; migration habitat is highly variable. |
| Piping Plover - coastal | 5 – Highly specific habitat used year-round. |
| Piping Plover - inland | 4 – Breeding habitat is somewhat flexible; wintering habitat is specialized. |
| Killdeer | 0 – Habitat throughout the year is variable. |
| Mountain Plover | 5 – Breeding habitat is specialized; habitats used throughout the remainder of the year are somewhat variable, but tend to use short grass habitat. |
| Spotted Sandpiper | 0 – Habitat throughout the year is variable. |
| Solitary Sandpiper | 4 – Breeding habitat is specialized; habitats used throughout the remainder of the year are highly variable. |
| Wandering Tattler | 3 – Breeding habitat is specialized; habitats used throughout the remainder of the year are highly variable. |
| Greater Yellowlegs | 4 – Breeding habitat is specialized; habitats used throughout the remainder of the year are highly variable. |
| Willet – eastern | 3 – The species uses a range of coastal habitats. |
| Willet – western | 3 – The species uses a range of interior habitats during breeding and migration and coastal habitats during the winter. |
| Lesser Yellowlegs | 3 – Breeding habitat is somewhat specialized; habitats used throughout the remainder of the year are highly variable. |
| Upland Sandpiper | 5 – The species uses a range of grassland habitats throughout the year. |
| Whimbrel | 3 – Breeding restricted to arctic and alpine tundra. |
| Bristle-thighed Curlew | 3 – Breeding habitat is moderately specialized; habitats used throughout the remainder of the year are somewhat variable. |
| Long-billed Curlew | 5 – Breeding habitat is highly specialized; habitats used throughout the remainder of the year are more variable. |
| Hudsonian Godwit | 0 – A range of habitats are used annually. Breeding habitat may be more specific, but there is concern we lack information about specific breeding requirements. |
| Bar-tailed Godwit | 5 – Breeding and wintering habitats are highly specialized. |
| Marbled Godwit | 5 – Breeding and wintering habitats are highly specialized. |
| Ruddy Turnstone | 5 – Breeding habitats less specialized than migratory and wintering habitats. |
| Black Turnstone | 5 – Breeding habitats less specialized than migratory and wintering habitats. |
| Red Knot | 4 – A range of coastal habitats is used. |
| Surfbird | 5 – Habitat used during breeding is different than habitats used during migration and the non-breeding season, but habitats used during each of the major portions of the annual cycle are very specific. |
| Stilt Sandpiper | 4 – Breeding habitat is specialized; habitats used throughout the remainder of the year are more variable. |
| Sanderling | 4 – Breeding habitat appears to be less specific than migratory habitat, but wintering habitat is almost exclusively sandy beaches. |
| Dunlin | 4 – Breeding habitat is specialized; habitats used throughout the remainder of the year are more variable. |
| Rock Sandpiper | 5 – Habitats used during the breeding season are slightly more varied than the strictly marine, coastal habitats used during migration and the non-breeding season. |
| Purple Sandpiper | 5 – The species uses rocky areas and shorelines year-round. |
| Baird's Sandpiper | 5 – Breeding and wintering habitats are highly specialized; migration habitat is highly variable. |
| Least Sandpiper | 3 – Breeding habitat is moderately specialized; habitats used throughout the remainder of the year are more variable. |
| White-rumped Sandpiper | 3 – Breeding habitat is moderately specialized; habitats used throughout the remainder of the year are more variable. |
| Buff-breasted Sandpiper | 4 – Breeding habitat is specialized; a different, specialized habitat is used during the non-breeding season. |
| Pectoral Sandpiper | 4 – Breeding habitat is specialized; habitats used throughout the remainder of the year are slightly more variable. |
| Semipalmated Sandpiper | 4 – Non-breeding habitat is most specific, and used habitats are more variable during migration and breeding. |
| Western Sandpiper | 3 – Breeding habitat is highly specialized; habitats used throughout the remainder of the year are more variable. |
| Short-billed Dowitcher | 3 – Breeding habitat is specialized; a different, specialized habitat is used during the non-breeding season and migration. |
| Long-billed Dowitcher | 3 – A range of habitats is used throughout the year. |
| Wilson's Snipe | 3 - This species is confined largely to wet grasslands and bogs but can tolerate relatively a wide variety of both natural and anthropogenic habitats. |
| American Woodcock | 4 - American Woodcocks are dependent on young forest habitat with nearby openings or fields during breeding season and migration. Habitat requirements during non-breeding season are more flexible but still require forest. |
| Wilson's Phalarope | 0 - Breeding and migratory habitats tend to be variable, and birds occurr over a wide range of habitat types during the non-breeding season and migration. |
| Red-necked Phalarope | 5 – Habitat is fairly specific throughout much of the annual cycle. |
| Red Phalarope | 5 - Breeding habitat is very specific; ocean habitat required and may use areas with specific conditions. |
