# Supplementary material for: Predicting Vulnerabilities of North American Shorebirds to Climate Change
Source: PLoS One. 2014 Sep 30;9(9):e108899. doi: 10.1371/journal.pone.0108899 (PMC4182597; doi:10.1371/journal.pone.0108899)
Supplement: Appendix S3 — Sensitivity analysis of risk category in which shorebirds are placed (DOC) [file pone.0108899.s003.doc]

**Galbraith et al.**

**Supplemental material**

|  | **Appendix 3** Sensitivity analysis of what USSCP risk category shorebirds would be in for a net 3, 4, 5, 6, and 7 arrows required to shift risk category; Table 2 uses 4 arrows. Risk categories are from the U.S. Shorebird Conservation Plan (USSCP): 1 = Not at Risk; 2 = Low Concern; 3 = Moderate Concern; 4 = High Concern; 5 = Highly Imperiled; we added another category at higher risk, 6 = Critical; parenthetic 7 emphasizes a sufficiently higher degree of risk that would result in an even higher risk category if one were created. | | | | | | | | |
| --- | --- | --- | --- | --- | --- | --- | --- | --- | --- |
|  | |  |  |  | Revised risk category with different amount of increased risk required to change categories | | | | |
| Species | | | Sum of ↑s, Table 2 | Current risk category | 7**↑** | 6**↑** | 5**↑** | 4**↑** | 3**↑** |
| Black-necked Stilt | | | 6 | 2 | 2 | 3 | 3 | 3 | 4 |
| American Avocet | | | 7 | 3 | 4 | 4 | 4 | 4 | 5 |
| American Oystercatcher | | | 8 | 4 | 5 | 5 | 5 | 6 | 6 |
| Black Oystercatcher | | | 5 | 4 | 4 | 4 | 5 | 5 | 5 |
| Black-bellied Plover | | | 8 | 3 | 4 | 4 | 4 | 5 | 5 |
| American Golden-Plover | | | 8 | 4 | 5 | 5 | 5 | 6 | 6 |
| Pacific Golden-Plover | | | 5 | 4 | 4 | 4 | 5 | 5 | 5 |
| Snowy Plover – coastal | | | 7 | 5 | 6 | 6 | 6 | 6 | 6(7) |
| Snowy Plover - inland | | | 6 | 5 | 5 | 6 | 6 | 6 | 6(7) |
| Wilson's Plover | | | 8 | 4 | 5 | 5 | 5 | 6 | 6 |
| Semipalmated Plover | | | 7 | 2 | 3 | 3 | 3 | 3 | 4 |
| Piping Plover – coastal | | | 8 | 5 | 6 | 6 | 6 | 6(7) | 6(7) |
| Piping Plover - inland | | | 7 | 5 | 6 | 6 | 6 | 6 | 6(7) |
| Killdeer | | | 0 | 3 | 3 | 3 | 3 | 3 | 3 |
| Mountain Plover | | | 3 | 5 | 5 | 5 | 5 | 5 | 6 |
| Spotted Sandpiper | | | 1 | 2 | 2 | 2 | 2 | 2 | 2 |
| Solitary Sandpiper | | | 7 | 4 | 5 | 5 | 5 | 5 | 6 |
| Wandering Tattler | | | 4 | 3 | 3 | 3 | 3 | 4 | 4 |
| Greater Yellowlegs | | | 4 | 3 | 3 | 3 | 3 | 4 | 4 |
| Willet – eastern | | | 7 | 3 | 4 | 4 | 4 | 4 | 5 |
| Willet – western | | | 5 | 3 | 3 | 3 | 4 | 4 | 4 |
| Lesser Yellowlegs | | | 4 | 3 | 3 | 3 | 3 | 4 | 4 |
| Upland Sandpiper | | | 3 | 4 | 4 | 4 | 4 | 4 | 5 |
| Whimbrel | | | 9 | 4 | 5 | 5 | 5 | 6 | 6 |
| Bristle-thighed Curlew | | | 6 | 4 | 4 | 5 | 5 | 5 | 6 |
| Long-billed Curlew | | | 5 | 5 | 5 | 5 | 6 | 6 | 6 |
| Hudsonian Godwit | | | 4 | 4 | 4 | 4 | 4 | 5 | 5 |
| Bar-tailed Godwit | | | 11 | 4 | 5 | 5 | 6 | 6 | 6(7) |
| Marbled Godwit | | | 5 | 4 | 4 | 4 | 5 | 5 | 5 |
| Ruddy Turnstone | | | 10 | 4 | 5 | 5 | 6 | 6 | 6(7) |
| Black Turnstone | | | 6 | 4 | 4 | 5 | 5 | 5 | 6 |
| Red Knot | | | 10 | 4 | 5 | 5 | 6 | 6 | 6 |
| Surfbird | | | 9 | 4 | 5 | 5 | 5 | 6 | 6(7) |
| Stilt Sandpiper | | | 6 | 3 | 3 | 4 | 4 | 4 | 5 |
| Sanderling | | | 11 | 4 | 5 | 5 | 6 | 6 | 6(7) |
| Dunlin | | | 8 | 3 | 4 | 4 | 4 | 5 | 5 |
| Rock Sandpiper | | | 4 | 3 | 3 | 3 | 3 | 4 | 4 |
| Purple Sandpiper | | | 3 | 2 | 2 | 2 | 2 | 2 | 3 |
| Baird's Sandpiper | | | 5 | 2 | 2 | 2 | 3 | 3 | 3 |
| Least Sandpiper | | | 5 | 3 | 3 | 3 | 4 | 4 | 4 |
| White-rumped Sandpiper | | | 6 | 2 | 2 | 3 | 3 | 3 | 4 |
| Buff-breasted Sandpiper | | | 7 | 4 | 5 | 5 | 5 | 5 | 5 |
| Pectoral Sandpiper | | | 6 | 2 | 2 | 3 | 3 | 3 | 4 |
| Semipalmated Sandpiper | | | 10 | 3 | 4 | 4 | 5 | 5 | 6 |
| Western Sandpiper | | | 7 | 4 | 5 | 5 | 5 | 5 | 6 |
| Short-billed Dowitcher | | | 7 | 4 | 5 | 5 | 5 | 6 | 6 |
| Long-billed Dowitcher | | | 7 | 4 | 5 | 5 | 5 | 5 | 6 |
| Wilson's Snipe | | | 2 | 3 | 3 | 3 | 3 | 3 | 3 |
| American Woodcock | | | 1 | 4 | 4 | 4 | 4 | 4 | 4 |
| Wilson's Phalarope | | | 4 | 4 | 4 | 4 | 4 | 5 | 5 |
| Red-necked Phalarope | | | 6 | 3 | 3 | 4 | 4 | 4 | 5 |
| Red Phalarope | | | 6 | 3 | 3 | 4 | 4 | 4 | 5 |
