# Supplementary material for: Predicting Vulnerabilities of North American Shorebirds to Climate Change
Source: PLoS One. 2014 Sep 30;9(9):e108899. doi: 10.1371/journal.pone.0108899 (PMC4182597; doi:10.1371/journal.pone.0108899)
Supplement: Appendix S4 — Species in each of the risk categories under the current system, and revised based on climate change. (DOC) [file pone.0108899.s004.doc]

**Galbraith et al.**

| **Supplemental material**  **Appendix 4** Species in each of the UCCSP risk categories under the current system, and revised based on climate change. Columns include requiring 3, 4, 5, 6, and 7 arrows to shift risk category. Risk categories are from the U.S. Shorebird Conservation Plan (USSCP): 1 = Not at Risk; 2 = Low Concern; 3 = Moderate Concern; 4 = High Concern; 5 = Highly Imperiled; we added an additional category at higher risk, 6 = Critical. | | | | | | |
| --- | --- | --- | --- | --- | --- | --- |
|  |  | Revised risk category with different amount of increased risk required to change categories | | | | |
| Risk Category | Current risk category | 7**↑** | 6**↑** | 5**↑** | 4**↑** | 3**↑** |
| Not at Risk (1) | ‒ | ‒ | ‒ | ‒ | ‒ | ‒ |
|  |  |  |  |  |  |  |
| Low Concern (2) | Semipalmated Plover | Black-necked Stilt | Spotted Sandpiper | Spotted Sandpiper | Spotted Sandpiper | Spotted Sandpiper |
|  | Black-necked Stilt | Spotted Sandpiper | Baird's Sandpiper | Purple Sandpiper | Purple Sandpiper |  |
|  | Spotted Sandpiper | White-rumped Sandpiper | Purple Sandpiper |  |  |  |
|  | White-rumped Sandpiper | Baird's Sandpiper |  |  |  |  |
|  | Baird's Sandpiper | Pectoral Sandpiper |  |  |  |  |
|  | Pectoral Sandpiper | Purple Sandpiper |  |  |  |  |
|  | Purple Sandpiper |  |  |  |  |  |
|  |  |  |  |  |  |  |
| Moderate Concern (3) | Black-bellied Plover | Semipalmated Plover | Semipalmated Plover | Semipalmated Plover | Semipalmated Plover | Killdeer |
|  | Killdeer | Killdeer | Killdeer | Killdeer | Killdeer | Baird’s Sandpiper |
|  | American Avocet | Wandering Tattler | Black-necked Stilt | Black-necked Stilt | Black-necked Stilt | Purple Sandpiper |
|  | Wandering Tattler | Greater Yellowlegs | Wandering Tattler | Wandering Tattler | White-rumped Sandpiper | Wilson’s Snipe |
|  | Greater Yellowlegs | Willet – western | Greater Yellowlegs | Greater Yellowlegs | Baird's Sandpiper |  |
|  | Willet – eastern | Lesser Yellowlegs | Willet – western | Lesser Yellowlegs | Pectoral Sandpiper |  |
|  | Willet – western | Least Sandpiper | Lesser Yellowlegs | Baird's Sandpiper | Wilson's Snipe |  |
|  | Lesser Yellowlegs | Rock Sandpiper | Least Sandpiper | White-rumped Sandpiper |  |  |
|  | Semipalmated Sandpiper | Stilt Sandpiper | White-rumped Sandpiper | Rock Sandpiper |  |  |
|  | Least Sandpiper | Wilson's Snipe | Rock Sandpiper | Pectoral Sandpiper |  |  |
|  | Rock Sandpiper | Red-necked Phalarope | Pectoral Sandpiper | Wilson's Snipe |  |  |
|  | Dunlin | Red Phalarope | Wilson's Snipe |  |  |  |
|  | Stilt Sandpiper |  |  |  |  |  |
|  | Wilson's Snipe |  |  |  |  |  |
|  | Red-necked Phalarope |  |  |  |  |  |
|  | Red Phalarope |  |  |  |  |  |
|  |  |  |  |  |  |  |
| High Concern (4) | American Golden-Plover | Black-bellied Plover | Black-bellied Plover | Black-bellied Plover | American Avocet | Semipalmated Plover |
|  | Pacific Golden-Plover | Pacific Golden-Plover | Pacific Golden-Plover | American Avocet | Wandering Tattler | Black-necked Stilt |
|  | Wilson's Plover | Black Oystercatcher | Black Oystercatcher | Willet – western | Greater Yellowlegs | Wandering Tattler |
|  | American Oystercatcher | American Avocet | American Avocet | Willet – eastern | Lesser Yellowlegs | Greater Yellowlegs |
|  | Black Oystercatcher | Willet – eastern | Willet – eastern | Upland Sandpiper | Willet – eastern | Willet – western |
|  | Solitary Sandpiper | Upland Sandpiper | Upland Sandpiper | Hudsonian Godwit | Willet – western | Lesser Yellowlegs |
|  | Upland Sandpiper | Bristle-thighed Curlew | Hudsonian Godwit | Least Sandpiper | Upland Sandpiper | Least Sandpiper |
|  | Whimbrel | Hudsonian Godwit | Marbled Godwit | Dunlin | Least Sandpiper | White-rumped |
|  | Bristle-thighed Curlew | Marbled Godwit | Semipalmated Sandpiper | Stilt Sandpiper | Rock Sandpiper | Sandpiper |
|  | Hudsonian Godwit | Black Turnstone | Dunlin | American Woodcock | Stilt Sandpiper | Pectoral Sandpiper |
|  | Bar-tailed Godwit | Semipalmated Sandpiper | Stilt Sandpiper | Wilson's Phalarope | American Woodcock | Rock Sandpiper |
|  | Marbled Godwit | Dunlin | American Woodcock | Red-necked Phalarope | Red-necked Phalarope | American Woodcock |
|  | Ruddy Turnstone | American Woodcock | Wilson's Phalarope | Red Phalarope | Red Phalarope |  |
|  | Black Turnstone | Wilson's Phalarope | Red-necked Phalarope |  |  |  |
|  | Surfbird |  | Red Phalarope |  |  |  |
|  | Red Knot |  |  |  |  |  |
|  | Sanderling |  |  |  |  |  |
|  | Western Sandpiper |  |  |  |  |  |
|  | Buff-breasted Sandpiper |  |  |  |  |  |
|  | Short-billed Dowitcher |  |  |  |  |  |
|  | Long-billed Dowitcher |  |  |  |  |  |
|  | American Woodcock |  |  |  |  |  |
|  | Wilson's Phalarope |  |  |  |  |  |
|  |  |  |  |  |  |  |
| Highly Imperiled (5) | Snowy Plover - coastal | American Golden-Plover | American Golden-Plover | American Golden-Plover | Black-bellied Plover | Black-bellied Plover |
|  | Snowy Plover - inland | Snowy Plover - inland | Wilson's Plover | Pacific Golden-Plover | Pacific Golden-Plover | Pacific Golden-Plover |
|  | Piping Plover - coastal | Wilson's Plover | American Oystercatcher | Wilson's Plover | Black Oystercatcher | Black Oystercatcher |
|  | Piping Plover - inland | Mountain Plover | Mountain Plover | American Oystercatcher | Mountain Plover | American Avocet |
|  | Mountain Plover | American Oystercatcher | Solitary Sandpiper | Black Oystercatcher | Bristle-thighed Curlew | Willet – eastern |
|  | Long-billed Curlew | Solitary Sandpiper | Long-billed Curlew | Mountain Plover | Hudsonian Godwit | Upland Sandpiper |
|  |  | Whimbrel | Whimbrel | Solitary Sandpiper | Marbled Godwit | Hudsonian Godwit |
|  |  | Long-billed Curlew | Bristle-thighed Curlew | Whimbrel | Black Turnstone | Marbled Godwit |
|  |  | Bar-tailed Godwit | Bar-tailed Godwit | Bristle-thighed Curlew | Semipalmated Sandpiper | Rock Sandpiper |
|  |  | Ruddy Turnstone | Ruddy Turnstone | Marbled Godwit | Western Sandpiper | Dunlin |
|  |  | Surfbird | Black Turnstone | Black Turnstone | Dunlin | Stilt Sandpiper |
|  |  | Buff-breasted Sandpiper | Surfbird | Surfbird | Solitary Sandpiper | Buff-breasted Sandpiper |
|  |  | Red Knot | Buff-breasted Sandpiper | Buff-breasted Sandpiper | Buff-breasted Sandpiper | Wilson's Phalarope |
|  |  | Sanderling | Red Knot | Semipalmated Sandpiper | Long-billed Dowitcher | Red-necked Phalarope |
|  |  | Western Sandpiper | Sanderling | Western Sandpiper | Wilson's Phalarope | Red Phalarope |
|  |  | Short-billed Dowitcher | Western Sandpiper | Short-billed Dowitcher |  |  |
|  |  | Long-billed Dowitcher | Short-billed Dowitcher | Long-billed Dowitcher |  |  |
|  |  |  | Long-billed Dowitcher |  |  |  |
| Critical (6) | ‒ |  |  |  |  |  |
|  |  | Snowy Plover - coastal |  | Snowy Plover - coastal | American Golden-Plover | American Golden-Plover |
|  |  | Piping Plover - coastal | Snowy Plover - coastal | Snowy Plover - inland | Snowy Plover - coastal | Snowy Plover - coastal |
|  |  | Piping Plover - inland | Snowy Plover - inland | Piping Plover - coastal | Snowy Plover - inland | Snowy Plover - inland |
|  |  |  | Piping Plover - coastal | Piping Plover - inland | Wilson's Plover | Wilson's Plover |
|  |  |  | Piping Plover - inland | Mountain Plover | Piping Plover - coastal | Piping Plover - coastal |
|  |  |  | Mountain Plover | Long-billed Curlew | Piping Plover - inland | Piping Plover - inland |
|  |  |  | Long-billed Curlew | Bar-tailed Godwit | American Oystercatcher | Mountain Plover |
|  |  |  |  | Ruddy Turnstone | Whimbrel | American Oystercatcher |
|  |  |  |  | Sanderling | Short-billed Dowitcher | Solitary Sandpiper |
|  |  |  |  | Red Knot | Long-billed Curlew | Whimbrel |
|  |  |  |  |  | Bar-tailed Godwit | Bristle-thighed Curlew |
|  |  |  |  |  | Ruddy Turnstone | Long-billed Curlew |
|  |  |  |  |  | Surfbird | Bar-tailed Godwit |
|  |  |  |  |  | Red Knot | Ruddy Turnstone |
|  |  |  |  |  | Sanderling | Black Turnstone |
|  |  |  |  |  |  | Surfbird |
|  |  |  |  |  |  | Red Knot |
|  |  |  |  |  |  | Sanderling |
|  |  |  |  |  |  | Semipalmated Sandpiper |
|  |  |  |  |  |  | Western Sandpiper |
|  |  |  |  |  |  | Short-billed Dowitcher |
|  |  |  |  |  |  | Long-billed Dowitcher |
